# Neural correlates of reward processing: Functional dissociation of two components within the ventral striatum

**DOI:** 10.1002/brb3.1987

**Published:** 2020-12-09

**Authors:** Filip Grill, Lars Nyberg, Anna Rieckmann

**Affiliations:** ^1^ Department of Radiation Sciences Umeå Center for Functional Brain Imaging Umeå University Umeå Sweden; ^2^ Department of Integrative Medical Biology Umeå University Umeå Sweden

**Keywords:** fMRI, functional connectivity, reward, ventral striatum

## Abstract

**Introduction:**

Rewarding and punishing stimuli elicit BOLD responses in the affective division of the striatum. The responses typically traverse from the affective to the associative division of the striatum, suggesting an involvement of associative processes during the modulation of stimuli valance. In this study, we hypothesized that fMRI responses to rewards versus punishments in a guessing card game can be disassociated into two functional component processes that reflect the convergence of limbic and associative functional networks in the ventral striatum.

**Methods:**

We used fMRI data of 175 (92 female) subjects from the human connectome project´s gambling task, working memory task, and resting‐state scans. A reward > punish contrast identified a ventral striatum cluster from which voxelwise GLM parameter estimates were entered into a k‐means clustering algorithm. The k‐means analysis supported separating the cluster into two spatially distinct components. These components were used as seeds to investigate their functional connectivity profile. GLM parameter estimates were extracted and compared from the task contrasts reward > punish and 2‐back > 0‐back from two ROIs in the ventral striatum and one ROI in hippocampus.

**Results:**

The analyses converged to show that a superior striatal component, coupled with the ventral attention and frontal control networks, was responsive to both a modulation of cognitive control in working memory and to rewards, whereas the most inferior part of the ventral striatum, coupled with the limbic and default mode networks including the hippocampus, was selectively responsive to rewards.

**Conclusion:**

We show that the fMRI response to rewards in the ventral striatum reflects a mixture of component processes of reward. An inferior ventral striatal component and hippocampus are part of an intrinsically coupled network that responds to reward‐based processing during gambling. The more superior ventral striatal component is intrinsically coupled to networks involved with executive functioning and responded to both reward and cognitive control demands.

## Graphical abstract

Using k‐means clustering, the striatal fMRI response to rewards can be separated into functional components.

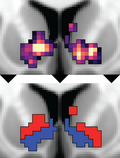

## 1 INTRODUCTION

The striatum subserves various aspects of behavior related to motor, associative, and affective processing and has been functionally divided into discrete divisions linked with these behaviors (e.g., Alexander et al., 1986; Tziortzi et al., 2014). The motor division of the striatum encompasses posterior dorsolateral portions of the putamen and caudate, while the affective division encompasses ventral striatum (VS), consisting of ventro‐medial caudate and putamen and nucleus accumbens (NAc); the associative division is situated in between the sensorimotor and affective division. Most of the striatum is morphologically homogenous consisting largely of GABAergic medium spiny neurons which receive widespread afferent connections from cortex and the dopaminergic midbrain. However, based on histology and connectivity, the NAc emerges as a relatively distinct area (Heimer et al., 1982) due to its afferent projections from limbic structures such as the amygdala and hippocampus as well as its efferent projections to the ventral pallidum which in turn sends projections to the dopaminergic midbrain (Haber, 2003).

The VS has been identified as an integral brain area for reward processing in mice, nonhuman primates, and humans (e.g., Haber & Knutson, 2010; Ikemoto & Panksepp, 1999; Salgado & Kaplitt, 2015). In human fMRI studies, the VS shows a differential response between rewarding and punishing stimuli, where rewards are associated with an increased blood oxygenation level dependent (BOLD) response when compared to punishments (e.g., monetary gains and losses, for review, see Knutson & Greer, 2008; Wang et al., 2016). The modulation of emotional valance is predominant in the affective division of the striatum but extends across the affective and associative divisions (e.g., Sescousse et al., 2013). Tasks that attempt to isolate BOLD responses to valence per se or to learning about rewards (i.e., associative properties) have shown largely overlapping response patterns (Fouragnan et al., 2018; Smith et al., 2016), suggesting that a functional division into affective and associative striatal compartments is oversimplified. Indeed, the extension across functional boundaries within the canonical reward response likely reflects the fact that cortico‐striatal connections are arranged in a continuous gradient rather than discrete subdivisions. A gradient‐like organization of cortico‐striatal connections has been shown in nonhuman primates (Haber & Knutson, 2010) and identified by resting‐state functional connectivity in humans (Marquand et al., 2017). A canonical reward response thus covers a portion of an affective‐cognitive gradient possibly combining aspects of behavior related to affective as well as associative processing (Haber, 2003; Haber et al., 2000; Haber & Knutson, 2010, for review).

Reward processing and dopamine functioning has long been intimately linked (Schultz, 1992, 2016; Wise, 1980). Animal work has suggested that projections from the hippocampus to the most inferior part of the VS play an important role in driving the responsivity of the dopamine system by releasing dopaminergic neurons from a nonfiring, inhibited state in behaviorally salient contexts (i.e., stimuli of potential value or importance are encountered; Floresco et al., 2001; Grace, 2012; Lisman & Grace, 2005). In human fMRI, it has been shown that hippocampus responds to high‐reward cues in a memory task (Adcock et al., 2006) and during reward prediction errors (e.g., Dickerson et al., 2011; Foerde & Shohamy, 2011). Moreover, the most inferior part of VS, corresponding approximately to NAc, is functionally connected to the central body of the hippocampus (Kahn & Shohamy, 2013). The strength of the connectivity between inferior VS and hippocampus has been shown to correlate with the density of striatal dopamine D2 receptors (Nyberg et al., 2016).

Considering both the absence of discrete functional boundaries in VS and its heterogeneous input, a large fMRI response to a modulation of stimuli valence thus might not reflect a functionally homogeneous “affective” response. Rather, it may reflect a locus of convergence of multiple circuits: an inferior ventral circuit involving interaction with the hippocampus and implicated in arousal of the dopaminergic system, and a second more superior ventral circuit that receives input from association cortex and may be predominantly involved in learning and executive control (i.e., the development and selection of appropriate responses and inhibition of inappropriate responses). This distinction has been shown in a resting‐state study that demonstrated differential patterns of connectivity within inferior and superior VS (Di Martino et al., 2008).

In the current study, we hypothesize that a seemingly homogenous fMRI response to rewards versus punishments in a guessing card game can be disassociated into two functional component processes by data‐driven clustering. We hypothesize that the different task components correspond with differential resting‐state functional connectivity profiles where an “affective component” shows connectivity with limbic structures including the hippocampus while an “associative component” will show connectivity with frontoparietal association networks. A region of interest (ROI) analysis was implemented to further investigate functional specificity of the components by comparing activation patterns across tasks (reward task versus executive control N‐back task). Based on our assumption that the superior, but not inferior, VS reflects an associative component process, we predicted that this ROI shows specific activation to a nonrewarded executive control N‐back task whereas the inferior VS does not.

To test our hypotheses, we used fMRI data from the human connectome project's (HCP) resting‐state, gambling task and working memory task from 175 healthy young participants. Being able to dissociate functional component processes of the neural response to rewards within the same contrast (reward > punishment) in VS contributes to an emerging view that the functions of human striatum are not accurately captured in terms of discrete functional compartments.

## 2 METHODS

### 2.1 Participants

The core sample consisted of 175 participants (92 female, mean age 28.8 [*SD* = 3.65]), mean years of education 14.95 [*SD* = 1.78]) from the HCP. Inclusion criteria consisted of having fully completed the gambling task, and participants were excluded if they made the same answer more than 80% of the trials (which could mean that they did not follow the task instructions). Participants had to have full resting‐state scans during the first day session. Participants’ EPI volumes were all reconstructed using the HCP “r227” algorithm. Participants were excluded if they had been marked by any quality control issue by the HCP consortium. The participants “mother identification” and “father identification” were unique to ensure that all participants were unrelated. 350 participants were identified that satisfied the inclusion and exclusion criteria. The final sample was split into a discovery and replication sample to give confidence to our results since the analysis pipeline involves several steps across various modalities with a focus on regions highly susceptible to artifacts.

### 2.2 Magnetic resonance imaging

Functional 3T EPI volumes that had gone through the “fMRIVolume Pipeline” (Glasser et al., 2013), from the gambling task, working memory task, and resting‐state EPI volumes were downloaded for each participant. Four of the core 175 participants did not have complete data for the working memory task and were thus excluded from the working memory analysis. A sample of 175 participants from the HCP, fulfilling the same inclusions criteria and matched with the core sample for gender, age, and education, was used to replicate the results (91 female, mean age 28.8 (*SD* = 3.73)), mean years of education 14.95 (*SD* = 1.75)). The study was approved by the local ethics committees at Umeå University, Umeå Sweden.

#### 2.2.1 fMRI data acquisition

The HCP MRI data acquisition is described in detail in Uğurbil et al. (2013). Briefly, fMRI was collected on a 3T TimTrio (Siemens) “Connectome” scanner with gradient‐echo EPI (spatial resolution 2 × 2 × 2 mm, 72 slices, TR = 720 ms, TE = 33.1 ms, flip angle = 52 deg, FOV = 208 × 180 mm, multiband factor = 8). For each subject, functional data were collected in two runs with orthogonal phase encoding (one left to right [LR] and one right to left [RL]).

#### 2.2.2 fMRI data preprocessing

All EPI volumes were processed through the HCP minimal processing pipeline (Glasser et al., 2013). Briefly, the EPI timeseries were motion corrected (6 DOF registration to a reference image), phase encoding distortion corrected, registered to the T1w structural image (6 DOF), and registered to the 2mm MNI template space. Additional preprocessing for the current study was carried out on the task fMRI including additional motion correction, spatial smoothing (FWHM = 4 mm), high‐pass temporal filter (0.024 Hz), and prewhitening. The resting‐state fMRI was additionally preprocessed including variance normalization by subtracting the mean divided by the standard deviation for each run (to allow for concatenation of a single subject's two runs), regressing out signal from deep white matter, regressing out signal from deep CSF, regressing out the global signal, spatial smoothing (FWHM = 4mm), and band‐pass temporal filter (low‐pass = 0.1 Hz, high‐pass = 0.01 Hz). Each subject's resting‐state fMRI runs were then concatenated (RL timepoints concatenated after LR timepoints).

### 2.3 Experimental design

#### 2.3.1 Gambling task

The gambling task involved a simple card‐guessing paradigm adapted from Delgado et al. (2000). Participants were shown a card with a question mark and were supposed to indicate whether the unseen number behind the question mark was above or below five. Correct guesses were rewarded with $1 during a feedback screen showing a green upwards pointing arrow. Incorrect guesses were punished with $−0.5 during a feedback screen showing a red downwards pointing arrow. If the number behind the question mark was neither above nor below five, a gray double‐headed arrow oriented vertically was shown during the feedback screen. All trials were pseudorandomized meaning that participant output did not change the feedback, or the resulting monetary reward given to them.

The trials were arranged in blocks with eight trials in each block. The blocks were either mostly rewarding (6/8 rewarding trials, where 2/8 were either a neutral or punishing trial) or mostly punishing (6/8 punishing trials, where 2/8 were either a neutral or rewarding trial). Each trial lasted for 3.5 s (question mark presentation and participant input 1.5 s, feedback 1 s, and 1 s intertrial interval) resulting in a block duration of 28 s. Each session consisted of two mostly rewarding and two mostly punishing blocks. A resting condition of 15 s was interleaved between each block. The duration for each gambling task run was 3:12 min. For more information regarding the task, see Barch et al. (2013).

#### 2.3.2 Working memory task

The working memory task consisted of a N‐back task with two levels of load and four categories of objects. The two levels of load were “2‐back” and “0‐back.” During the 2‐back condition, participants were shown a train of stimuli and were asked to indicate whether the current stimulus is the same as the stimulus presented two steps back. During the 0‐back condition, a target stimulus was presented, and participants were then shown a train of stimuli and asked to indicate if the current stimulus was the same as the target stimulus presented at the start. Thus, the 2‐back condition entailed constantly updating the current target stimulus while 0‐back entailed maintaining a single target stimulus. Each session of the task consisted of eight blocks (four 2‐back, four 0‐back) and within each block one of four possible stimulus categories were presented (faces, places, tools, body parts).

At the start of each block, a 2.5 s cue indicated the load level as well as presenting the target if the condition was 0‐back. Each block consisted of ten trials, and each trial consisted of a 2.5 s stimulus presentation and an intertrial interval of 0.5 s. Each run had four fixation blocks, each with a duration of 15 s positioned after every other block. The duration for each working memory task run was 5:01 min. For more information regarding the task, see Barch et al. (2013).

### 2.4 Statistical analysis

#### 2.4.1 Gambling task GLM

A general linear model (GLM), implemented through FSL’s FEAT (Woolrich et al., 2001), was used to estimate BOLD signal related to rewards and punishments during the task in a first level individual analysis. Two regressors were defined for each individual run (LR and RL separately), one for the (mostly) reward blocks and one for the (mostly) punish blocks, six regressors of residual movement‐related artifacts (three rigid‐body translations and three rotations computed from the motion correction) were included in the design as regressors of no interest. The resting condition served as an implicit baseline. The reward condition effects were contrasted against the punish condition effects (reward > punish). To merge the results from the individual runs (LR and RL), the parametric maps were taken to a second level fixed effects analysis within each participant.

To identify a group level reward response in the VS the second level contrast maps for reward > punish were taken to a random effects group analysis and corrected using threshold free cluster enhancement (TFCE) through FSL’s randomise function (Winkler et al., 2016) with 5,000 sign‐flip permutations. Cortical activations are not reported.

#### 2.4.2 Separating the reward response signal

A k‐means clustering algorithm was used to investigate the hypothesis that the seemingly homogenous VS reward response can be disassociated into component parcels. Briefly, by minimizing the variance within and maximizing the variance between a set of k clusters, the k‐means clustering algorithm determines whether a distribution of values are better described as two or more groups rather than a single group of (homogenous) response pattern across all voxels in the VS cluster. The k‐means algorithm was given each voxel reward parameter estimate on the group level as one dimension and each voxel punish parameter estimate as a second dimension taken from the voxels inside the significant VS reward > punish cluster. Note that no spatial priors were given in the analysis. The optimal number of clusters was determined using the average silhouette method between *k*(1, 2, 3, 4, 5). After each voxel was assigned to a cluster, the data were projected back into volumetric space yielding separable parcels of the initial reward > punish cluster.

#### 2.4.3 Seed‐based resting‐state functional connectivity

**FIGURE 1:**
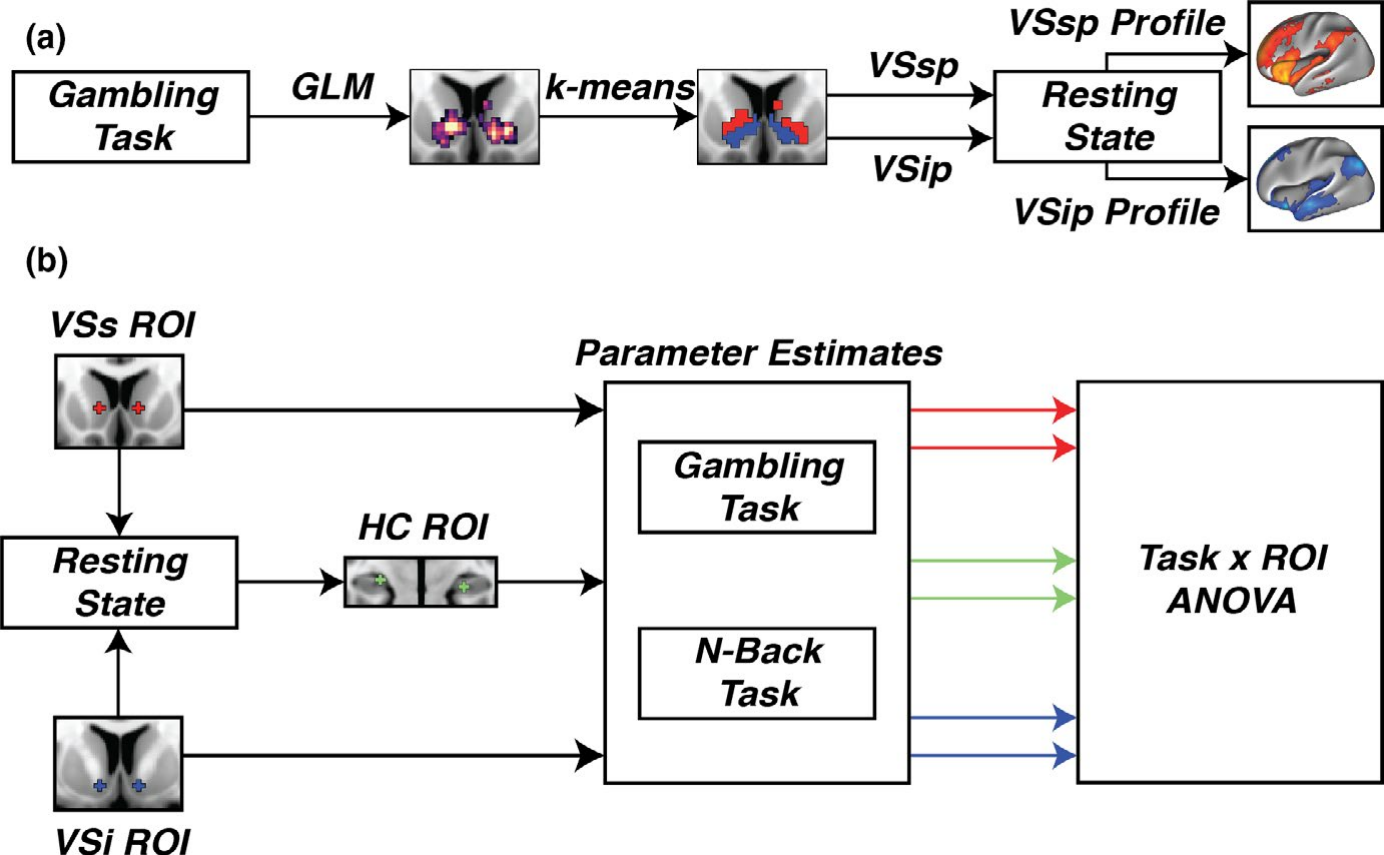
(a) Parcellation pipeline. A GLM was used to find a cluster in the ventral striatum using the contrast reward > punish. The reward parameter estimates and the punish parameter estimates for each voxel in the cluster were taken to a k‐means clustering algorithm. The k‐means algorithm identified separate parcels that were used as seeds in a resting‐state functional connectivity analysis. (b) ANOVA pipeline. Seed ROIs (VSs and VSi) from Di Martino et al., 2008 were used to find the maximum functional connectivity between VSi and the hippocampus. The maximum connectivity was used to define a ROI in the hippocampus (HC ROI). Parameter estimates from the two contrasts (reward > punish and 2‐back > 0back) were extracted from each ROI. The parameter estimates were then taken to a task x ROI ANOVA

The resulting parcels from the k‐means analysis were used as seeds in a resting‐state functional connectivity analysis. The mean time series of each parcel was correlated against each voxel in the brain using Pearson's correlation to yield functional connectivity maps on an individual level. The resulting functional connectivity maps were r‐to‐z transformed. The individual maps were then entered into a random effects group level analysis using FSL’s randomize with 5,000 sign‐flip permutations and TFCE correction. Only positive correlations were considered in the analysis. The group t‐statistic maps from the resting‐state functional connectivity analysis were projected to the fsaverage surface, included in the Freesurfer distribution (Fischl, 2012), for visualization purposes and to calculate dice similarity coefficients (DSC). In order to identify which functional network a parcel mostly corresponded to, DSC were calculated between the 7‐network resting‐state parcellation, provided in fsaverage surface space by Yeo et al. (2011), and the cortical functional connectivity map for each parcel. These analysis steps are depicted in Figure 1a.

#### 2.4.4 Region of interest definition

To further investigate the hippocampal connectivity with the VS and the functional specificity of the components, we defined two regions of interest (ROIs). Two VS ROIs were defined from MNI coordinates described in the literature, VSi (±10, 8, −8), and VSs (±10, 14, 0) first reported by Di Martino et al. (2008). These ROIs were used as initiating seeds in a resting‐state functional connectivity analysis (i.e., a voxelwise computation of the correlation between seed and target timeseries). The resulting whole‐brain functional connectivity maps were r‐to‐z transformed. A contrast between the ROIs was created on the individual level subtracting the z‐statistic maps of the VSs from the VSi (VSi > VSs). A hippocampal ROI was then defined using the maximum difference of the contrast in a one sample *t* test on the group level. Each ROI consisted of bilateral spheres 3mm in radius (27 voxels for each individual ROI). The reason for defining regions of interest were twofold: 1) to balance the number of voxels used as seeds in the functional connectivity analysis and later extraction of parameter estimates from areas in close proximity; 2) to show that the functional dissociation in the data‐driven analysis corresponds to loci that are commonly used and cited in the literature (e.g., Di Martino et al., 2008; Gabbay et al., 2013; Harrison et al., 2009; Nyberg et al., 2016; Sarpal et al., 2015).

#### 2.4.5 Working memory task GLM

A GLM, implemented through FSL’s FEAT (Woolrich et al., 2001), was used to estimate BOLD signal related to 2‐back and 0‐back (ignoring the stimulus category) during the task in a first level individual analysis. Two regressors were defined for each individual run (LR and RL separately), one for the 2‐back blocks and one for the 0‐back blocks. The 2‐back condition effects were contrasted against the 0‐back condition effects (2‐back > 0‐back). To merge the results from the individual runs (LR and RL), the parametric maps were taken to a second level fixed effects analysis within each participant.

#### 2.4.6 Task x ROI ANOVA

To investigate the hypothesis that an associative component will show an increased BOLD response to cognitive load while an affective component will not, we extracted the participant's parameter estimates from the contrasts reward > punish and 2back > 0back from the three ROIs. The extracted parameter estimates were then entered into a 2x3 ANOVA to establish that VSi, VSs, and HC differ in their responses across tasks. A post hoc investigation was then made by looking at the means and confidence intervals of each contrast and ROI. These analysis steps are depicted in Figure 1b.

#### 2.4.7 Replication analyses

Statistical analyses were repeated in the replication sample. Pearson´s correlation coefficient was calculated between the two samples’ parcel‐based functional connectivity parametric maps to compare how well the parcels of the two samples connected to the same areas.

## 3 RESULTS

### 3.1 Data‐driven analysis shows a separable reward response in the VS

**FIGURE 2:**
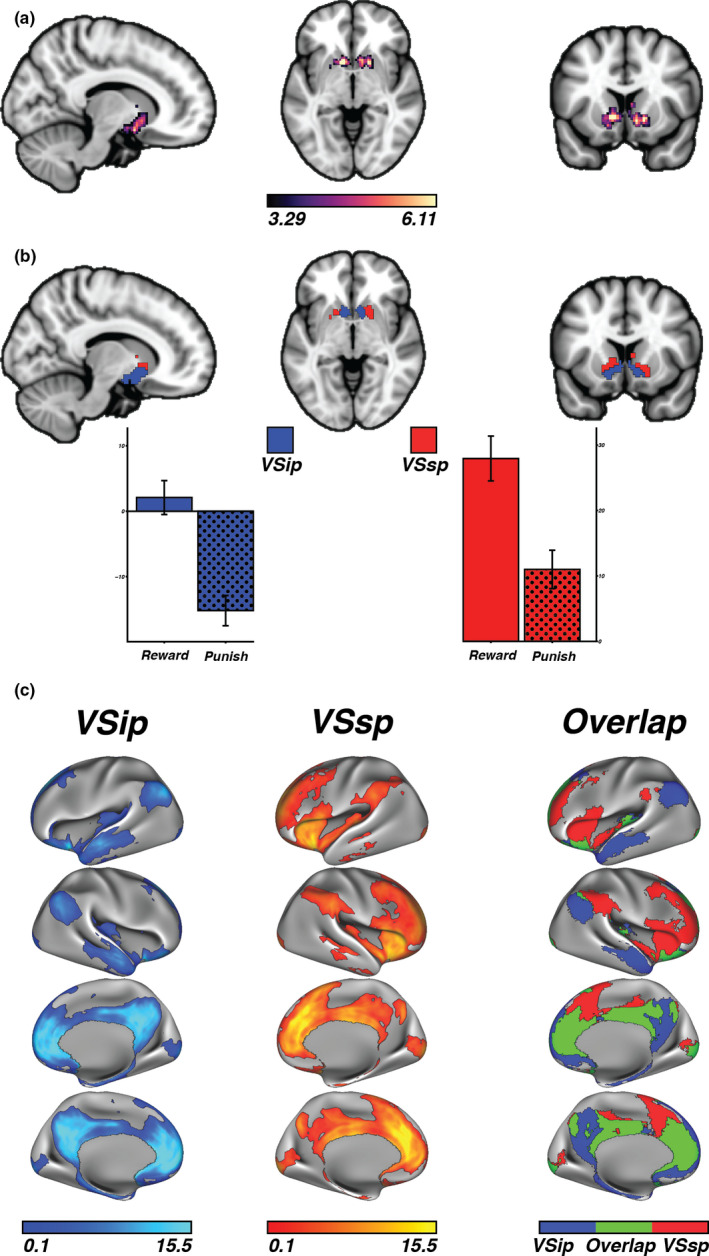
(a) The initial reward > punish cluster in the ventral striatum. Legend represents *t* statistics. (b) The two parcels, VSip and VSsp given by the k‐means clustering algorithm. Extracting the parameter estimates from reward and punish, respectively, showed that the VSip reward > punish difference was driven by less negative BOLD response to reward while the VSsp was driven by differences in positive BOLD responses to both conditions. (c) Functional connectivity profiles of the parcels. The VSip was predominantly connected to the default mode network, while the VSsp showed a split connection to the ventral attention and frontal control network. Spatial overlap between the parcel's functional connectivity profiles (threshold *p* < .05, respectively) was predominantly observed in the medial prefrontal cortex and posterior cingulate cortex

In line with prior studies and meta‐analyses (e.g., Sescousse et al., 2013), the voxelwise analysis of the gambling task revealed a large single‐cluster reward (>punishment) response in VS that includes both NAc and caudate (*p* < .0005, TFCE corrected cluster size 722 voxels; Figure 2a). Nevertheless, when mean responses of each of the gambling task conditions for each voxel in the VS cluster were entered into a k‐means clustering algorithm, the analysis showed an optimal solution of k = 2 (Table 1). This suggests that the VS cluster is better described in terms of two segregated parcels. Visual inspection of the k‐means clustering suggests one inferior part and one superior part, here referred to as the VS inferior parcel (VSip) and the VS superior parcel (VSsp), respectively (Figure 2b). The mean parameter estimates of reward and punish, respectively, in VSip showed that the observed delta in the reward > punish contrast was driven by less negative BOLD response to reward while the VSsp was driven by differences in positive BOLD responses to both conditions (VSip reward *M* = 1.78, CI = [−0.81 to 4.37]; VSip punish *M* = −15.55, CI = [−17.88 to −13.22]; VSsp reward *M* = 27.46, CI = [24.06–30.86]; VSsp punish *M* = 10.58, CI = [7.68–13.48]; Figure 2b).

**TABLE 1:** Average silhouette width per number of clusters from the *k*‐means analysis on the mean parameter estimates of reward and punish for each voxel in the VS cluster. A solution for two clusters shows the largest average silhouette width and was deemed the optimal solution

*K* number of clusters				
1	2	3	4	5
0.00	0.57	0.49	0.48	0.49

### 3.2 Functional connectivity confirms differential network profiles

VSip and VSsp were separately used as seeds in a resting‐state functional connectivity analysis conducted on the whole brain to understand network similarities and differences between the parcels. The analysis showed spatial overlap between the parcel's functional connectivity in medial prefrontal cortex and posterior cingulate cortex. However, connectivity to limbic areas including hippocampus was constrained to the VSip whereas connectivity to anterior insula and temporoparietal junction (i.e., parts of what is known as the ventral attention network) was constrained to the VSsp. The VSip was predominantly connected to the default mode network, while the VSsp showed a split connection to the ventral attention and frontal control network (Table 2; Figure 2c).

**TABLE 2:** Dice scores between whole brain cortical resting‐state connectivity and Yeo et al. (2011) cortical parcellation using VSip and VSsp as separate seeds. Limbic areas are mostly contained in VSip, while ventral attention areas are mostly contained in VSsp. Overlap can be seen in the frontal control and default mode network; however, VSsp mostly correspond to the frontal control network while VSip mostly correspond to the default mode network

	Resting‐State Network						
	Visual	Somato‐Motor	Dorsal Attention	Ventral Attention	Limbic	Frontal Control	Default Mode
VSip	0.07	0.04	0.00	0.03	0.16	0.15	0.68
VSsp	0.07	0.03	0.00	0.38	0.05	0.39	0.29

### 3.3 Dissociation of task responses in a priori defined resting‐state regions of interest

**FIGURE 3:**
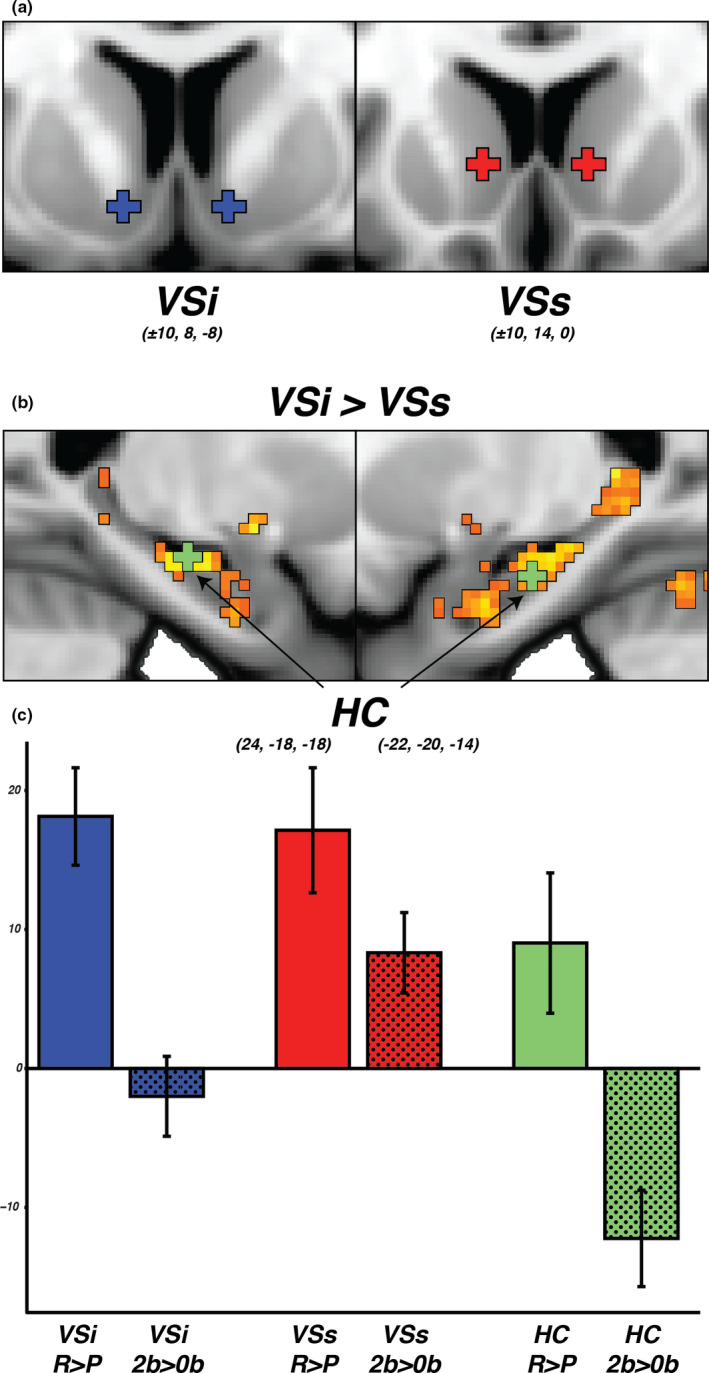
(a) Location of ROIs from Di Martino et al. (2008); VSi and VSs. (b) Location of hippocampus ROI at the strongest connectivity between VSi and hippocampus (HC). All coordinates are in MNI space. (c) Parameter estimates from each ROI and contrast that were used in the ANOVA. All three ROIs show an increased BOLD response to reward > punish (R>P), while only the VSs show an increased BOLD response to 2‐back > 0‐back (2b>0B). The dissociated response pattern for VSs indicates that this area is recruited during increased cognitive load while the VSi and HC are not. The VSs is also recruited during rewarding stimuli which together suggest that the VSs might be involved in more associative aspects of reward processing

The functional parcellation into VSip and VSsp corresponds well to the distinction of inferior and superior striatum ROIs (VSi and VSs; Figure 3a) previously described by Di Martino et al. (2008). Replicating this prior work (Di Martino et al., 2008; Nyberg et al., 2016), the resting‐state functional connectivity map of the VSi seed showed stronger coupling with central hippocampus than VSs (maximum difference in connectivity in central hippocampus right: *x* = 24, *y* = −18, *z* = −18, *t*‐stat = 3.65; left: *x* = −22, *y* = −20, *z* = −14, *t*‐stat = 4.09; Figure 3b). Along with VSi and VSs, the bilateral hippocampal peak coordinates were included as a ROI in subsequent task analyses. For each of the three ROIs, parameter estimates were extracted from the gambling and working memory task. An ANOVA showed a significant main effect of task (*F*(1, 1,032) = 41.94, *p* < .001), a significant main effect of ROI (*F*(2, 1,032) = 19.39, *p* < .001), and a significant interaction between task and ROI (*F*(2, 1,032) = 4.39, *p* < .05) confirming a dissociation of task response across ROIs. Post hoc comparisons illustrated in the bar graph in Figure 3c show that the results confirm our prediction of a joint response of the VSi and the functionally connected central hippocampus during reward processing (VSi: reward > punish, mean = 18.13, CI = [14.62–21.64]; HC: reward > punish, mean = 9.02, CI = [3.97–14.07]) but not in a task taxing executive functioning (VSi: 2back > 0back, mean = −2.00, CI = [−4.87 to 0.87]; HC: 2back > 0back, mean = −12.23, CI = [−15.70 to −8.76]). In contrast, VSs responded significantly above zero for rewarded blocks in the gambling task as well as to higher demands in the working memory task (VSs: reward > punish, mean = 17.14, CI = [12.64–21.64]; 2back > 0back, mean = 8.32, CI = [5.42–11.22]).

### 3.4 Replication of the findings

Since the analysis pipeline involved several steps, and because the partitioning of a given sample's task response cluster into functional components of VS was purely data driven, we thought it important to demonstrate that the k‐means solution and subsequent results were not sample specific. For this reason, we repeated all analyses in a second HCP sample of 175 individuals. The reward > punish contrast in the replication sample also showed a significant response in the VS (*p* < .0005, TFCE corrected, cluster size 752 voxels). The k‐means analysis performed on the VS cluster in the replication sample showed an optimal solution of *k* = 2, which again suggested a separable response pattern contained in the cluster corresponding to the VSi and VSs parcels. The voxelwise correlation of the VSip functional connectivity parametric maps between the original and replication sample showed a Pearson correlation of 0.94. The voxelwise correlation of the VSsp parametric maps between the original and replication sample showed a Pearson correlation of 0.96, indicating an excellent correspondence between the two samples. The functional connectivity contrast between VSi and VSs in the replication sample also showed a maximum difference in connectivity in central hippocampus, with great spatial congruency (right: *x* = 24, *y *= −20, *z* = −14, *t*‐stat = 6.86; left: *x* = −22, *y* = −20, *z* = −14, *t*‐stat = 7.24). The left peak voxel thus had the same coordinate in both samples, while the right peak voxel differed 4 mm in the *y*‐direction and 8 mm in the *z*‐direction. Extracting the parameter estimates from the two contrasts replicated the original finding (VSi: reward > punish, mean = 14.30, CI = [10.57–18.03]; 2back > 0back, mean = −2.58, CI = [−5.52 to 0.36]; VSs: reward > punish, mean = 18.80, CI = [14.28–23.32]; 2back > 0back, mean = 8.74, CI = [5.83–11.65]; HC: reward > punish, mean = 10.30, CI = [4.50–16.10]; 2back > 0back, mean = −22.60, CI = [−27.21 to −17.99]).

## 4 DISCUSSION

Prior fMRI studies have demonstrated a reliable and extensive response to rewards in the VS. In this study, we show that the reward response in the VS consists of a mixture of heterogenous signals that are integrated into a single‐cluster BOLD response on the level of a GLM contrast. Two distinct signals that are spatially separable along an inferior to superior division could be dissociated. An affective component in the inferior VS was functionally connected to limbic areas, including an area of the hippocampus that also showed a BOLD response to rewards. The affective component was not positively modulated by a task taxing executive functioning, indicating that the modulation seen during rewards was not a task general response but specific to stimuli with positive valance. An associative component situated dorsal to the affective component was functionally connected to attention and control areas (e.g., anterior cingulate and lateral prefrontal cortex) and was positively modulated by both rewards as well as by increased working memory load when comparing response patterns across two functional tasks. Together, these findings suggest that a “typical” response to rewards covers parts of an affective‐cognitive VS gradient and can be separated into component processes.

### 4.1 Two‐component response to rewards

Prior resting‐state studies have suggested a functional distinction of inferior and superior VS such that VSi is coupled more strongly to orbitofrontal cortex and VSs with, for example, lateral prefrontal cortex (Di Martino et al., 2008), reflecting the fact that VS is not functionally homogeneous and separated by a sharp boundary from associative striatum but instead may be better understood as an affective‐cognitive gradient (Marquand et al., 2017). The current study extends these findings to show that the VSi connectivity profile is further distinguished from VSs by its selective connectivity and coactivation with hippocampus. Models of dopamine release posit that hippocampus subiculum regulates the responsiveness of striatal dopaminergic neurons in response to behavioral salience (Floresco et al., 2001; Grace, 2012; Lisman & Grace, 2005). The intrinsic VS‐hippocampal coupling might thus reflect a functional circuit involved in an affective response that provides a salience signal to the most inferior part of VS and releases dopamine neurons from a nonfiring state into a “state of alert.” Indeed, the hippocampal ROIs identified in the current study correspond with the approximate location of the subiculum. Even though disinhibition and activation cannot be disentangled with fMRI, it is noteworthy in this context that the inferior VS response to rewards was driven by a less negative BOLD signal as compared to an implicit baseline, maybe suggesting a less inhibited state, whereas the superior VS response was driven by a difference in positive BOLD signal.

The observation that only the superior VS showed a response to a taxing cognitive control task (2‐back modulation in working memory) suggests that this area is part of an associative component. In the context of the gambling task, the response may reflect the evaluation of the outcome of choices made during the task even when those aspects of the task are not explicitly taxed here. This is in line with previous observations of a superior VS rather than inferior VS response to reward prediction errors, that is, when updating the current value of stimulus response relationships (e.g., Ballard et al., 2019). This inferior to superior division is consistent with the idea of an affective‐cognitive gradient of the reward system (Haber, 2003; Marquand et al., 2017), where rewards are processed in a ventral to dorsal gradient with increasing cognitive demand.

In the whole‐brain resting‐state functional connectivity analysis, we further confirmed that separable parcels in VS exhibited different, but partly overlapping, network profiles, as would be expected from nearby areas in an inherently gradient‐like structure. In terms of distinct network connectivity, the inferior parcel was predominantly connected to limbic as well as to areas associated with the default mode network. Our interpretation of this component's relation to affective processing is supported by studies linking the default mode network with basic skin conductance response in humans (Fan et al., 2012) as well as to the fraction of eyelid opening in nonhuman primates (Chang et al., 2016), suggesting a relationship between the default mode network and arousal. The superior VS parcel was connected to frontal control and ventral attention networks, which are implicated in working memory and attentional demands. Its functional connections to these networks together with the area's specific response to a modulation of working memory load suggest a clear role of superior VS in cognitive control. Interestingly, a strong overlap between the parcels’ functional connectivity profiles was observed in the medial prefrontal cortex. The medial prefrontal cortex has long been established as a central area for reward processing, value‐based decision‐making, and emotional regulation (for review see Hiser & Koenigs, 2018). Recent research in rodents has shown that increased excitability in the medial prefrontal cortex reduces striatal response and inhibits behavioral drive for dopaminergic stimulation (Ferenczi et al., 2016), thus acting in an antagonistic relationship to the hippocampal modulation of dopaminergic neurons. The medial prefrontal cortex is then well positioned to integrate information carried by the affective and associative component into more complex representations which can influence the striatal dopamine system. Also, task dependent functional connectivity between the striatum and areas of the prefrontal cortex have been shown to be modulated by affective and informative reward properties suggesting that distinct reward properties are processed through interactions between the prefrontal cortex and striatum (Smith et al., 2016). Potential future work should incorporate a task design that can distinguish the modulation of connectivity between the striatum and prefrontal cortex depending on affective and associative reward processes.

### 4.2 Potential significance for psychopathologies

Aberrant reward processing has been identified in various psychopathologies such as major depressive disorder, bipolar disorder, and schizophrenia which possibly relate to symptoms common across the conditions (Whitton et al., 2015), for example, negative affective symptoms and cognitive symptoms. Indeed, a blunted VS BOLD response to rewards is observed in individuals with major depressive disorder (Ng et al., 2019) as well as in individuals with schizophrenia (Gradin et al., 2013) which might relate to anhedonia and abnormal dopamine functioning (Lamontagne et al., 2018). Given the separation of a simple reward response into component processes, it is possible that, depending on psychopathology or symptomology, one or both components exhibit a blunted reward response. A blunted reward response in the affective component might relate to anhedonia, while a blunted reward response in the associative component might relate to cognitive symptoms. However, due to the looping cortico‐striatal connections, it is also possible that a blunted reward response in the affective component influences the response in the associative component or vice versa, resulting an overall diminished reward response. Unfortunately, in the current study, a sample of healthy young adults were investigated and the components correlation with psychiatric traits could not be addressed.

### 4.3 Limitations

There are a number of limitations to the current study. Most importantly, while we consider it a strength to be able to compare fMRI responses across three different states (rest, rewards, cognitive control) in a large sample of individuals, the task design does not allow for firm conclusions regarding the component processes of reward in VSs and VSi. This is in part due to the fact that the gambling task at hand did not lend itself to disentangle reward anticipation from reward prediction errors. A strategy for future work to separate component processes might involve dissociating VS responses to reward prediction errors from a more general reward response. Relatedly, other aspects of the comparison between tasks, like general task complexity, difficulty or novelty, could not be controlled in this study. Thus, the interaction effect of task differences in BOLD response observed in the ANOVA may reflect for example increasing levels of task complexity rather than affective versus associative demands (i.e., working memory versus a simple guessing game). Note, however, that for the reward task at least, task complexity is controlled *within task* by the nature of the task contrast reward > punish. Nevertheless, the ANOVA reveals a response in both ROIs for this contrast, which we find difficult to reconcile with an explanation by which the dissociation across ROIs is driven by task complexity alone.

Finally, because of tissue boundaries, the VS is a region with relatively low signal to noise ratio (Choi et al., 2012). It is therefore possible that an inferior to superior signal intensity gradient is picked up by the k‐means algorithm. It is, however, unlikely that signal intensity artifacts conform to create parcels that follow known anatomical striatal organization (Tziortzi et al., 2014) and that the parcels functionally connect to distinct cortical networks.

### 4.4 Conclusions

In prior work, the fMRI response to rewards (versus punishment) has been thought to reflect the function of the affective striatum. In this study, we show that the fMRI response to rewards in the VS actually consists of a mixture of signals, likely reflecting component processes of reward. We show that the inferior component and hippocampus are part of an intrinsically coupled network that responds to reward‐based processing during gambling but not to a task involving cognitive control. According to prominent animal models, the inferior VS and hippocampus are part of a functional circuit responsible for controlling midbrain dopaminergic responsiveness and we propose that the observed BOLD response and functional coupling could reflect a human analogue governed by this model. The component located more superior was intrinsically coupled to networks involved with executive functioning and attention and responded to a task involving cognitive control. We speculate that this component is related to processing cognitive aspects of reward evaluation. Our findings have potential implications for psychiatric research since it might be feasible to disentangle affective and cognitive contributions to a blunted reward response seen across various psychopathologies.

## CONFLICT OF INTEREST

The authors declare that there is no conflict of interest.

## AUTHOR CONTRIBUTION

FG, LN, and AR contributed to the design of the study, interpretation of the results, writing and revising the manuscript. FG performed the data analysis.

### Peer Review

The peer review history for this article is available at https://publons.com/publon/10.1002/brb3.1987.

## Data Availability

The data that support the findings of this study are available from the Human Connectome project, WU‐Minn Consortium. Restrictions apply to the availability of these data, which were used under license for this study. Data are available https://dp.humanconnectome.org with the permission of the Human Connectome project, WU‐Minn Consortium. Neuroimaging data on the group level can be viewed and downloaded from https://neurovault.org/collections/9039/ . Other supporting data are available upon reasonable request from the corresponding author.
